# A novel homozygous *PSAP* mutation identified by whole exome sequencing in a consanguineous family with metachromatic leukodystrophy: a case report

**DOI:** 10.1177/03000605241301877

**Published:** 2024-11-29

**Authors:** Xueyi Li, Xiaoni Kuang, Guangwen Huang, Zhenyu Liu, Shuyuan Yan

**Affiliations:** 1 Changsha Hospital for Maternal & Child Health Care of Hunan Normal University; 2 Hunan Provincial Maternal and Child Health Care Hospital; 3 Hunan Provincial People’s Hospital/The First Affiliated Hospital of Hunan Normal University

**Keywords:** Metachromatic leukodystrophy, *PSAP*, lysosome, mutation, whole exome sequencing, Sanger sequencing, arylsulfatase A, sphingolipid, neurodegeneration

## Abstract

Metachromatic leukodystrophy (MLD) is a genetic lysosomal disease. Here, we investigated the role of prosaposin (*PSAP*) gene mutations in MLD. This current case report describes a female patient who presented with motor development regression at two years and five months of age. The symptoms included difficulty walking, loss of ambulation, increased muscle tension, limb pain, and intentional tremors. Brain magnetic resonance imaging revealed potential white matter lesions, while electromyography indicated neurogenic damage in both lower limbs. Gesell assessment showed severe motor retardation, along with mild retardation in adaptability, speech, and social communication. Whole exome sequencing analysis identified a homozygous mutation in the *PSAP* gene, specifically c.643A>G, resulting in the amino acid change p.N215D. Immunofluorescence assays of cultured cells indicated no impact on the PSAP protein lysosomal localization, but the mutation was associated with a decreased lysosomal pH and reduced cathepsin D activity. Transmission electron microscopy revealed changes in lysosome morphology and abnormal protein aggregation. These findings suggest that the *PSAP* c.643A>G (p.N215D) mutation may be a causal factor for MLD in this patient. This discovery may provide new insights into the genetic basis and pathophysiological mechanisms of MLD.

## Introduction

Metachromatic leukodystrophy (MLD) is a type of lysosomal storage disorder that affects sphingolipid metabolism and is classified as both leukodystrophy and sphingolipidosis.^1^ MLD is characterized by neurodegeneration with progressive demyelination.^2^ It is most commonly associated with a mutation in the *ARSA* gene,^3^ which is mapped to chromosome 22 (22q13.33). *ARSA* encodes arylsulfatase A, the enzyme responsible for the breakdown of certain types of fat, called sulfatides, in cells. The *ARSA* gene mutation results in an enzyme that is deficient or dysfunctional, leading to a buildup of sulfatides in various tissues, particularly the nervous system. This sulfatide accumulation causes gradual damage to the peripheral nerves and white matter of the brain, which is characteristic of MLD.

In addition to the *ARSA* gene, mutations in the prosaposin (*PSAP*) gene are associated with MLD. *PSAP* encodes the prosaposin protein, which is a precursor for four small proteins: saposin A, B, C, and D. Saposins are activators of certain fat metabolism-related enzymes, including arylsulfatase A.^4,5^ Because saposin B stimulates arylsulfatase A activity,^6^ a mutation in the *PSAP* gene may give rise to dysfunctional saposin B that would subsequently impair arylsulfatase A activity. This could occur even with wild-type (WT) arylsulfatase A, resulting in the characteristic sulfatide buildup that leads to the damage observed in MLD.^7,8^ MLD cases resulting from *PSAP* mutations are usually referred to as saposin B deficiency or “variant forms” of MLD. Some specific *PSAP* mutations have also been linked to MLD, including point mutations and splice site mutations.^9,10^

It should be noted that reports of *PSAP* mutations related to MLD are relatively rare, and more genetic evidence is needed. Functional studies on these mutations are also required to confirm their relationship with MLD. Here, we report a case of MLD in a Chinese family caused by a novel c.643A>G *PSAP* mutation in a female patient. Through *in vitro* experiments, we also found that this c.643A>G (p.N215D) mutation does not affect the protein localization in lysosomes, but can cause abnormal lysosome aggregation and dysfunction.

## Materials and methods

### Inclusion and exclusion criteria

For this *ex vivo* study, which was informed by a detailed case report, certain inclusion and exclusion criteria were applied when selecting patient samples. The following inclusion criteria were used: (1) the patient was diagnosed with MLD, (2) sufficient biological samples were available for *ex vivo* analysis, and (3) informed consent was obtained from the patient or legal representative. The following exclusion criteria were applied: (1) the samples were compromised from contamination or inadequate handling, (2) the patient had concurrent conditions that could confoundingly affect the study outcomes, and (3) a lack of essential clinical or pathological information necessary for interpreting the results.

### Family ascertainment

Because of the consanguinity, it was presumed that the inheritance pattern in this family followed an autosomal recessive pattern. The research included four family members: the proband, her parents (who were unaffected), and her brother.

### DNA extraction

Peripheral blood samples were collected from the subject, her parents, and her brother after receiving informed consent. Genomic DNA was extracted from peripheral blood leukocytes using the DNeasy Blood & Tissue Kit (Qiagen, Hilden, Germany) following the manufacturer’s suggested protocol.

### Whole exome sequencing (WES)

After fragmenting 1.5 μg of genomic DNA with a Covaris S2 Ultrasonicator (Covaris, Woburn, MA, USA), as recommended by the manufacturer, Agilent SureSelect libraries were generated for the WES analysis of family members II-1 and II-3. The exome capture was performed using the SureSelect Human All Exon V5 kit (Agilent, Santa Clara, CA, USA) for 51 Mb. The paired-end sequencing of barcoded exome libraries was performed on the HiSeq 4000 platform (Illumina, San Diego, CA, USA) using 150-bp + 150-bp reads. Sequences were paired demultiplexed and aligned to the human genome (NCBI build 37/hg19) by Burrows and Wheeler alignment.^11^ Further analyses were performed using the Genome Analysis Toolkit (GATK), SAMtools, and Picard, with GATK Unified Genotyper employed for variant calling. Variant annotation was performed with Ensembl release 82 and ANNOVAR Documentation was used to implement filtering using various single nucleotide polymorphism (SNP) databases, such as dbSNP (build 138; http://www.ncbi.nlm.nih.gov/projects/SNP/index.html), Exome Variant Server (release ESP6500SI-V2; http://evs.gs.washington.edu/EVS/), 1000 Genomes Project (released May 2012; http://www.1000genomes.org/), and HapMap CHB (release #28; http://hapmap.ncbi.nlm.nih.gov/). To predict the possible impact of variants, SIFT (http://sift.jcvi.org/), Polyphen-2 (http://genetics.bwh.harvard.edu/pph2/), Mutation-Taster (http://www.mutationtaster.org/), and Human Splicing Finder (http://www.umd.be/HSF3/) were used.

### Sanger sequencing

The candidate variants were recognized using Sanger sequencing. Primers were designed using Primer Premier 5.0 to amplify fragments containing the variant with the following sequences: forward, 5′-CCAGAGCTGGACATGACTGA-3′; reverse, 5′-TTCTTAATGGGCTCCACCAG-3′. The PCR products underwent sequencing using the ABI PRISM 3730 DNA Analyzer (Applera Corporation, Norwalk, CT, USA) and the ABI PRISM Big-Dye Terminator Cycle Sequencing v.3.1 Ready Reaction Kit.

### Protein structural and functional analysis

To assess the structural consequences of *PSAP* mutations on the PSAP protein, we used the Swiss-Model software. Initially, we selected the appropriate templates using sequence similarity, coverage, and resolution. The WT PSAP sequence was retrieved from UniProt, then the mutations were introduced using the mutagenesis tool within Swiss-Model. The mutated sequences were then submitted for structure prediction in the Automated Mode. The resulting models were evaluated for quality, then optimized and compared with the WT structure using PyMOL. Furthermore, we conducted a functional impact analysis of the mutations using SIFT, Polyphen-2, and Mutation-Taster to obtain a comprehensive understanding of their effects on PSAP.

### Cell culture

Cells were maintained in an appropriate culture medium supplemented with necessary growth factors and antibiotics at 37°C in a humidified incubator with 5% CO_2_.

### Plasmid construction and transfection

*PSAP* cDNA was obtained from Origene (Rockville, MD, USA) and cloned into the pCMV-3xFLAG vector (Sigma-Aldrich, St. Louis, MO, USA). The QuikChange mutagenic kit (Stratagene, La Jolla, CA, USA) was used to generate the *PSAP* p.N215D mutant. To transfect the *PSAP*-Flag plasmid into cells, Lipofectamine 2000 (Invitrogen, Thermo Fisher Scientific, Waltham, MA, USA) was used as the transfection reagent. Briefly, cells were plated at a density suitable for transfection and allowed to adhere overnight. The *PSAP*-Flag plasmid was then transfected into the cells following the manufacturer’s suggested protocol to ensure optimal transfection efficiency.

### Immunofluorescence staining

Cultured cells were washed twice with phosphate-buffered saline (PBS), then fixed with 3.7% paraformaldehyde for 10 minutes at room temperature. Subsequently, the cells were permeabilized with 0.1% Triton X-100 for 10 minutes at room temperature. Next, the cells were blocked with 5% bovine serum albumin for 30 minutes at room temperature, then incubated with the primary antibody (anti-Flag, clone M2, Sigma-Aldrich) at a 1:500 dilution overnight at 4°C. An Alexa Fluor 488-conjugated secondary antibody (Invitrogen) was used for detection (1:1000 dilution) and incubated for 1 hour at room temperature in the dark. The sample was examined using a confocal microscope (TCS SP5; Leica, Wetzlar, Germany), which was equipped with a Plan-Apochromat 63×NA 1.4 oil differential interference contrast objective.

### Lysosome staining

Lysosomes in cells were visualized using LysoSensor™ Green DND-189 (Invitrogen). Cells were seeded on coverslips, allowed to adhere, and then treated or transfected as previously described. Subsequently, the cells were incubated with 1 μM LysoSensor™ Green DND-189 for 30 minutes at 37°C in a humidified incubator with 5% CO_2_. After PBS washes, the cells were fixed with 4% paraformaldehyde and mounted onto glass slides. DAPI was used for nuclear counterstaining. Images were captured using a confocal microscope, then fluorescence intensity was quantified using ImageJ software (National Institutes of Health, Bethesda, MD, USA). The data are presented as a percentage of the fluorescence intensity relative to the WT group.

### Cathepsin D activity assay

A cell lysate-based assay was used to examine cathepsin D activity (ab65302, Abcam, Cambridge, MA, USA) following the manufacturer’s protocol. Fluorescence intensity was measured with an excitation wavelength of 328 nm and an emission wavelength of 460 nm using a fluorometer (Thermo Fisher Scientific). The data are presented as a percentage of fluorescence intensity relative to the WT cohort.

### Transmission electron microscopy (TEM)

The cells were fixed with 2.5% glutaraldehyde overnight at 4°C. Following fixation, all specimens were post-treated with 2% OsO4 (Electron Microscopy Sciences, Hatfield, PA, USA). The specimens were dehydrated using a series of fractional acetone, then immersed in a mixture of acetone and resin (1:1). Following this, they were embedded in Ebon (Sigma-Aldrich). A slide containing an ultra-thin section (70 nm) was doubly stained using lead nitrate (supplied by Sigma-Aldrich) and uranyl acetate (provided by Ted Pella, Redding, CA, USA). A Tecnai G2 Spirit transmission electron microscope (FEI Company, Hillsboro, OR, USA) and Eagle 4k HS digital camera (FEI Company) were used to capture the images.

## Results

### Clinical report

At the time of the report, the female proband was 2 years and 5 months of age. She was born on time after a trouble-free pregnancy and normal delivery. She was diagnosed with motor development regression 7 months prior to the report. She began walking independently at 1 year and 2 months old, began to trot at 1 year and a half, and was able to speak short sentences by 1 year and 9 months of age. However, from about 1 year and 10 months old, her motor development began to regress. She started having difficulty walking, gradually losing her ability to walk, then experienced increased muscle tension in both lower limbs, limb pain, and developed intentional tremors in both upper limbs. Over time, her language capabilities and cognitive development decreased.

**Figure 1.**
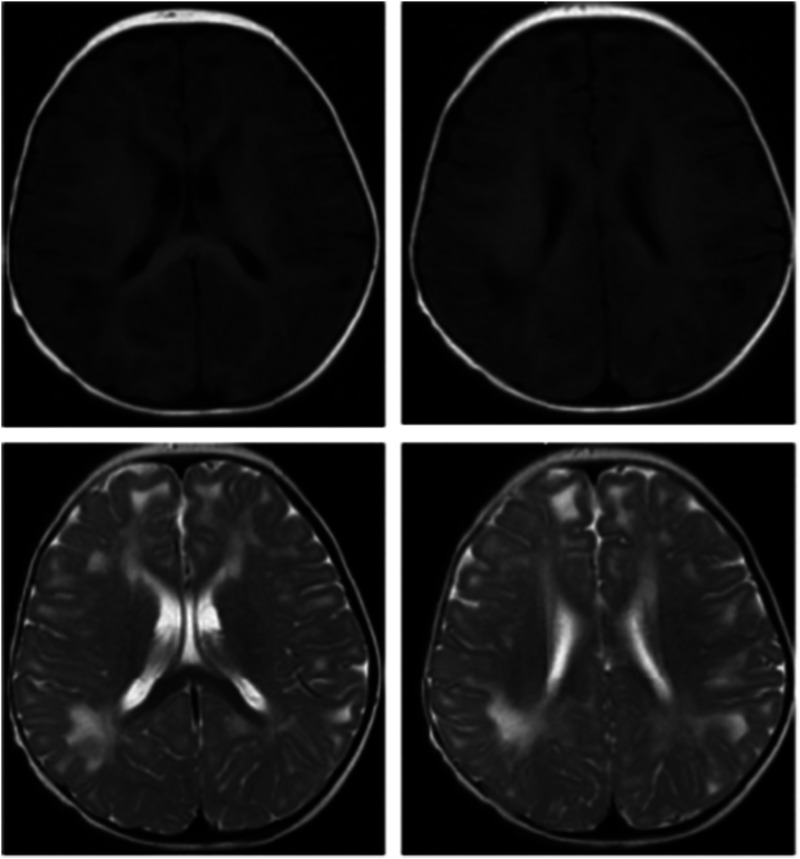
Brain magnetic resonance imaging (MRI) scan of the proband. The axial image using T2-weighting demonstrates bilaterally high-intensity white matter that is abnormal in the centrum semiovale. This image displays a tigroid pattern with a radial arrangement of hypointense stripes, clearly indicated by the arrows. Importantly, the white matter of the subcortical areas remains unaffected. The FLAIR image in the axial plane displays an extensive region of hyperintense white matter within the centrum semiovale. FLAIR, fluid-attenuated inversion recover.

Upon hospital admission, a brain magnetic resonance imaging (MRI) scan showed diffuse long T1 and long T2 shadows in the bilateral white matter, indicating potential white matter lesions (Figure 1). Electromyography revealed a slowed conduction velocity in the bilateral common peroneal nerve and a low amplitude of evoked potential. The conduction speed of the motor nerves of the bilateral tibial nerves was also decreased, suggesting neurogenic damage in both lower limbs. Auditory evoked potentials showed that bilateral wave intervals III–IV were greater than those of I–III. A Gesell development assessment indicated a motor score of 32, severe growth retardation, and mild growth retardation in adaptability, speech ability, and social communication.

The patient’s seven-year-old brother exhibited a similar history. He displayed significant spasticity in his limbs, was unable to walk or sit independently, and experienced severe mental and language retardation. As a result, he was unable to care for himself.

Molecular analysis revealed the same homozygous c.643A>G variant in the elder brother, indicating that he also had the same disease.

### Molecular analysis

**Figure 2.**
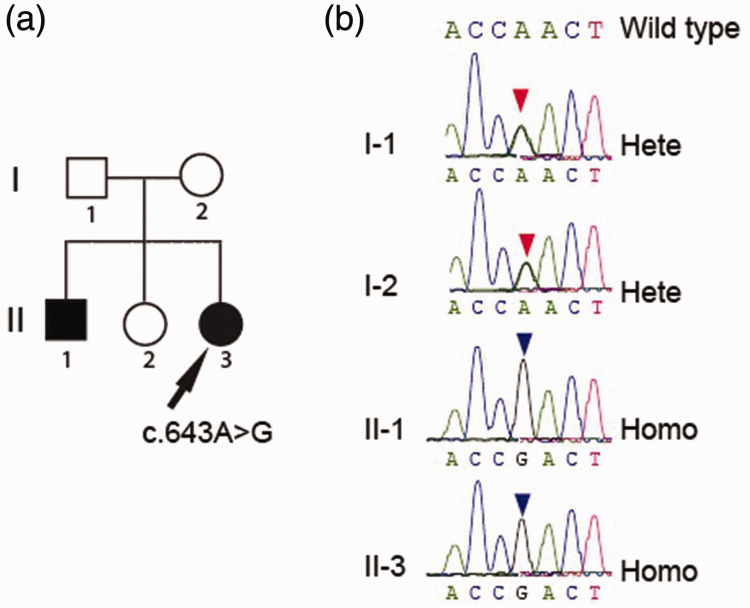
A novel pathogenic prosaposin (*PSAP*) gene mutation in a Chinese family with metachromatic leukodystrophy (MLD). (a) The family pedigree for *PSAP*, where an empty square signifies a healthy male and an empty circle denotes a healthy female. Conversely, a solid circle indicates a female patient and a solid square represents a male patient. The proband is highlighted by the arrow abd (b) sanger sequencing confirmed the pathogenic *PSAP* variant (c.643A > G) is present in the proband (II-3), her parents (I-1, I-2), and her brother (II-1).

Figure 2(a) displays the family’s pedigree. Targeted sequencing revealed a homozygous c.643A>G mutation within *PSAP* (NM_001042465.3: c.643A>G, p.N215D) in the proband (II-3) and her brother (II-1), which was subsequently verified using Sanger sequencing. The mutation was also confirmed by Sanger sequencing to be inherited from the parents (I-1 and I-2) (Figure 2).

**Figure 3.**
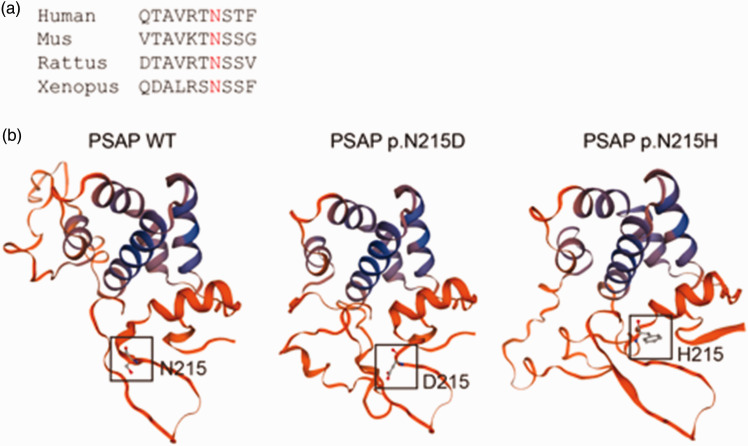
Crystal structure of the prosaposin (PSAP) protein with mutated amino acids. (a) The multi-species PSAP protein sequence alignment generated using ClustalX2 software. The affected amino acid is marked in red and (b) the crystal structure of PSAP amino acids 198–263 (wild-type PSAP). The impacts of the p.N215D mutation and previously reported p.N215H mutation on PSAP protein structure are predicted. The positions of the mutated amino acids are highlighted by white boxes.

We submitted these mutation data to the Leiden Open Variation Database (LOVD). The individual ID is #00377058. The p.N215 *PSAP* protein is conserved among various species (Figure 3(a)).

Using Swiss-Model software, we predicted the structural effects of the p.N215D mutation on the PSAP protein. Additionally, we predicted the effects of the p.N215H mutation, another amino acid change in the same codon. This mutation has been previously reported in a Spanish ancestry family by Wood et al. (2000) and is classified as pathogenic in ClinVar.^12^ The results showed that p.N215D and p.N215H have a greater impact on the non-helical structure of PSAP. The p.N215D and p.N215H mutants show more structural similarity compared with the WT PSAP protein (Figure 3(b)).

### PSAP p.N215D induces lysosomal abnormalities

**Figure 4.**
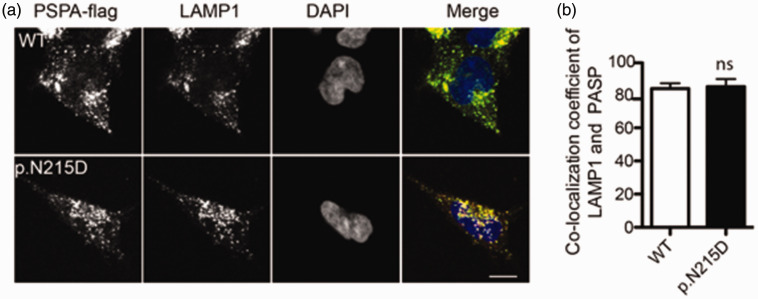
Lysosomal localization of the p.N215D prosaposin (PSAP) mutation. (a) HeLa cells transfected with either pCMV-PSAP-Flag, a plasmid encoding wild-type (WT) PSAP, or a PSAP mutant (p.N215D) were co-immunostained with p.N215D anti-Flag (green) and anti-LAMP1 (red) antibodies. The scale bar represents 10 μm and (b) a statistical graph of the colocalization of WT PSAP and p.N215D PSAP mutant with the lysosomal marker protein LAMP1.

To explore the effect of the p.N215D mutation on PSAP function, we constructed a mutant expression plasmid and overexpressed it in mammalian cells. The results suggested that the p.N215D mutation had no impact on PSAP distribution, which was correctly localized in the lysosomes (Figure 4).

**Figure 5.**
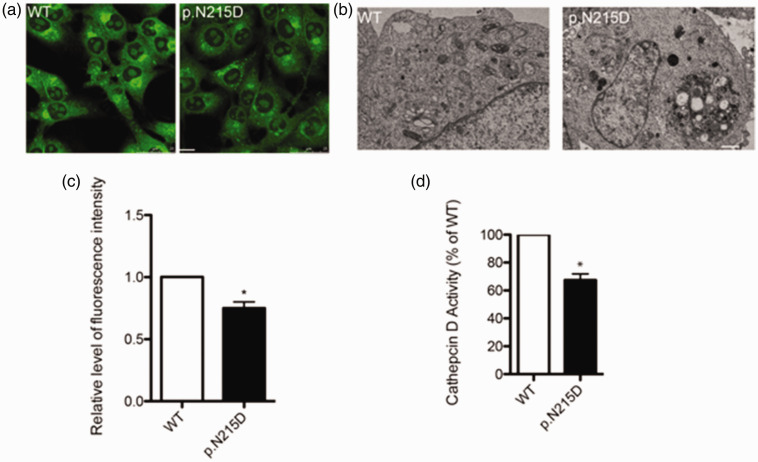
The p.N215D prosaposin (PSAP) mutation induces lysosomal abnormalities. (a) The lysosomes in cells overexpressing wild-type (WT) PSAP or the p.N215D mutant were stained with LysoSensor™ Green DND-189. The scale bar denotes 10 μm. (b) Transmission electron microscopy analysis of the lysosomes in HeLa cells expressing WT PSAP or the p.N215D mutant. Representative images of damaged lysosomes (indicated by white arrows) are displayed. The scale bar signifies 1 μm. (c) The fluorescence intensity statistics from panel a and (d) cathepsin D activity levels after the cells were transfected with either WT PSAP or the p.N215D mutant. *P < 0.05.

It should be noted that lysosome staining with pH-sensitive LysoSensor™ dyes showed that the lysosomal pH decreased in cells overexpressing the p.N215D mutant compared with WT PSAP (Figure 5(a,c)). TEM results also showed that the p.N215D mutation caused abnormal lysosome accumulation in the cells (Figure 5(b)). More importantly, the PSAP p.N215D mutant resulted in decreased cathepsin D activity in lysosomes compared with WT PSAP, suggesting impaired lysosomal function (Figure 5(d)).

## Discussion

The *PSAP* gene encodes prosaposin, which is processed to yield four smaller proteins referred to as saposins (A, B, C, and D). Each saposin is an essential co-factor for different enzymes involved in the metabolism of certain lipids within lysosomes.^13,14^ Lysosomes are the recycling centers of the cell that break down various types of waste molecules, including lipids. Saposins A and C are required for decomposing sphingolipids,^15,16^ saposin B is required for galactosylceramide and sulfatide decomposition,^17,18^ and saposin D is involved in glycosylceramide hydrolyzation.^19^ Mutations in the *PSAP* gene can cause dysfunction of one or more saponins, impairing the associated lysosomal enzyme(s) and leading to a buildup of specific lipids within lysosomes. This process underlies several types of lysosomal storage disorders, including MLD and Gaucher disease,^20^ which are characterized by neurodegeneration and other systemic symptoms.^21^ *PSAP* gene mutations, particularly those affecting the coding region within the B domain of saposin, can lead to sulfate accumulation and result in rare MLD variants with normal arylsulfatase A activity.^22^ Saposins all originate from the same precursor, prosaposin, in the late endosome/lysosome and can regulate the lysosomal enzymatic degradation of various sphingolipids. Therefore, saponins have a notable impact on sphingolipid pathogenesis, with this group of lysosomal storage diseases identified by sphingolipid buildup.^23,24^ According to current data from the Human Gene Mutation Database, fewer than 15 mutations in the *PSAP* gene have been identified in MLD.^22,25^

This study is the first to report a case in a Chinese family where clinical and neuroradiological features of the proband led to a suspected diagnosis of MLD. Genetic testing was pursued directly without prior measurement of leukocyte arylsulfatase A enzymatic activity. Although it has been documented that arylsulfatase A enzymatic activity may not always correlate with the MLD phenotype,^26^ examining this factor remains valuable when studying MLD and *PSAP* mutations. However, our experimental investigations focused on the *PSAP* gene, demonstrating a new homozygous missense variant (c.643A > G) indicative of the rare MLD variant associated with saposin B deficiency. While mutations at the c.643A site have been previously reported in MLD,^12^ our case involves different mutated amino acids. In our instance, the mutation resulted in a change from asparagine (N) to aspartic acid (D) at position p.N215. This position in the *PSAP* gene is conserved across various species.

PSAP protein is primarily localized to the lysosomes, but evidence has suggested that it is present in other cellular organelles and locations. For example, PSAP has been identified in the Golgi body and endoplasmic reticulum,^3^ which are involved in protein synthesis, modification, and transportation, which is consistent with its role in the cell. PSAP can also be secreted outside of cells, where it can act as a neurotrophic factor influencing cell survival, differentiation, and myelination.^3^ PSAP can bind to the sortilin receptor, which is also a binding partner of progranulin (PGRN). When PSAP is bound to sortilin, it prevents PGRN from binding to the same receptor and being internalized and degraded in the lysosome. This leads to increased PGRN levels outside of the cell.^27^ PGRN has also been linked to certain neurodegenerative conditions, including Alzheimer’s disease and frontotemporal dementia.^28,29^ In the current study, the p.N215D mutation did not alter the lysosomal localization of PSAP. However, whether it affects the PSAP distribution in other organelles or extracellular secretion remains to be further studied. Moreover, the mechanisms and physiological significance of this extracellular role are not as well understood as its intracellular functions. Given the abnormal lysosome accumulation observed via TEM and decreased cathepsin D activity, our data suggest that the p.N215D mutation can impact PSAP functionality.
